# Raman spectroscopy on dried blood plasma allows diagnosis and monitoring of colorectal cancer

**DOI:** 10.1002/mco2.774

**Published:** 2024-10-31

**Authors:** Carlo Morasso, Elena Daveri, Arianna Bonizzi, Marta Truffi, Francesco Colombo, Piergiorgio Danelli, Sara Albasini, Licia Rivoltini, Serena Mazzucchelli, Luca Sorrentino, Fabio Corsi

**Affiliations:** ^1^ Laboratory of Nanomedicine Istituti Clinici Scientifici Maugeri IRCCS Pavia Italy; ^2^ Translational Immunology Unit Fondazione IRCCS Istituto Nazionale dei Tumori di Milano Milan Italy; ^3^ Division of General Surgery “Luigi Sacco” University Hospital ASST Fatebenefratelli‐Sacco Milan Italy; ^4^ Department of Biomedical and Clinical Sciences University of Milan Milan Italy; ^5^ Breast Unit Istituti Clinici Scientifici Maugeri IRCCS Pavia Italy; ^6^ Colorectal surgery unit Fondazione IRCCS Istituto Nazionale dei Tumori di Milano Milan Italy

**Keywords:** biomarkers, colorectal cancer, dried blood plasma, inflammation, Raman spectroscopy

## Abstract

Colorectal cancer (CRC) remains challenging to diagnose, necessitating the identification of a noninvasive biomarker that can differentiate it from other conditions such as inflammatory bowel diseases (IBD) and diverticular disease (DD). Raman spectroscopy (RS) stands out as a promising technique for monitoring blood biochemical profiles, with the potential to identify distinct signatures identifying CRC subjects. We performed RS analysis on dried plasma from 120 subjects: 32 CRC patients, 37 IBD patients, 20 DD patients, and 31 healthy controls. We also conducted longitudinal studies of CRC patient's postsurgery to monitor the spectral changes over time. We identified six spectral features that showed significant differences between CRC and non‐CRC patients, corresponding to tryptophan, tyrosine, phenylalanine, lipids, carotenoids, and disulfide bridges. These features enabled the classification of CRC patients with an accuracy of 87.5%. Moreover, longitudinal analysis revealed that the spectral differences normalized over 6 months after surgery, indicating their association with the presence of the disease. Our study demonstrates the potential of RS to identify specific biomolecular signatures related to CRC. These results suggest that RS could be a novel screening and monitoring tool, providing valuable insights for the development of noninvasive and accurate diagnostic methods for CRC.

## Graphical abstract

Blood serves as a valuable repository of disease biomarkers, offering insights for diagnosis. Raman spectroscopy (RS) emerges as a promising technique, capable of discerning distinct molecular signatures in biological samples. This study demonstrates RS's efficacy in distinguishing dried plasma samples across various bowel diseases

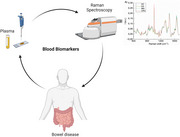

## 1 INTRODUCTION

Several patients each year complain colorectal‐related symptoms, such as hematochezia, stypsis, tenesmus, or lower quadrants pain.^1^, ^2^, ^3^ Upfront colonoscopy is the gold standard to rule out organic diseases, such as colorectal cancer (CRC), inflammatory bowel diseases (IBD), or complicated diverticular disease (DD).^4^, ^5^, ^6^ However, colonoscopy is an invasive procedure with potential complications and discomforts for patients. These complications are quite rare but not negligible, with a rate of perforation after screening colonoscopy up to 0.08%, and a bleeding risk of 1%, especially if polypectomy is needed.^7^ Nevertheless, a twofold higher risk of bleeding was observed in patients undergoing colonoscopy after positive fecal immunochemical test.^8^ Furthermore, it should be noted that endoscopic procedures might carry a higher risk of complications, up to 6.8% in patients aged >75 years old.^9^ ^−12^Interestingly, only 0.4–1.1% of colonoscopies performed for the above‐mentioned symptoms reveal a CRC and about 18.3% are positive for colorectal adenomas, while the great majority of the procedures are basically negative.^13^ These data become even more relevant in the setting of screening colonoscopies in asymptomatic patients with a positive occult blood test, since these endoscopic procedures are performed in a larger number of subjects compared with symptomatic patients.^8^ Therefore, healthcare costs and complications‐related burden are heavy, while CRC is found with a relatively low rate.^14^ Thus, a noninvasive, specific blood biomarker to detect CRC, possibly discriminating other colorectal diseases such as IBD and DD, would be helpful to properly address those patients to colonoscopy. Not only diagnosis, but also disease monitoring after treatments is a concern for CRC. Indeed, standard follow up after surgery and adjuvant treatments relies on imaging and repeated colonoscopies, but blood‐derived features that promptly indicate CRC recurrence are still lacking. Tumor‐related serum markers, such as carcinoembryonic antigen (CEA) or carbohydrate antigen 19.9 (Ca19.9) are currently used in standard clinical practice, but their sensitivity ranges from 29 to 64%,^15^, ^16^ and their expression at baseline is widely heterogeneous, thus questioning their usefulness in follow up after CRC resection. More recently, liquid biopsy has been explored in clinical trials to accurately monitor CRC after surgery and even to decide about adjuvant treatments irrespectively from pathologic staging.^17^ However, costs and the need to characterize the primary tumor by next‐generation sequencing to build the specific probe are still severely limiting its wide adoption in routine clinical practice.^18^

Raman spectroscopy (RS) is an advanced optical technique that draws unique chemical molecular signature of biological samples.^19^, ^20^ The key aspect of RS is the coupling of the photons of an incident light to specific vibrational modes of the molecule, resulting in transitions between the quantised vibrational states, and inelastic scattering of the photons, whose spectrum reflects the structure of the molecules illuminated.^19^ The main strength of RS is that this approach does not require any particular sample preparation or chemical labeling simplifying the analysis. This reduces the chances of introducing artifacts or contamination. RS also saves time and resources as no reagent and consumable is required. Additionally, RS provides real‐time information about the sample, making it suitable for rapid analysis and disease process monitoring for a number of disorders.^21^, ^22^

In the context of cancer diagnosis, RS can provide valuable information of the molecular species present in the blood, their structure and their relative amount. By analyzing the Raman spectra of dried plasma samples, specific biomarkers or spectral features associated with certain diseases can be identified. This can potentially aid in the detection and monitoring of disease progression^23^ in different types of cancer,^24^, ^25^ neurodegenerative,^26^, ^27^ and metabolic disorders.^28^, ^29^, ^30^ The correlation between Raman spectra and plasmatic levels of CEA has also been recently reported.^31^ However, the available literature is mainly based on comparisons between two populations (usually healthy subjects (healthy controls [HCs]) and CRC patients), and there are no data on the effectiveness of the method in distinguishing between IBD and DD from CRC.^32^ Additionally, to the best of our knowledge, there are no published studies on the spectroscopic analysis of blood samples posttreatment to demonstrate the association between observed spectral differences and the presence of cancer.

The aim of this study was thus to investigate the Raman fingerprint of CRC by comparing it with different colorectal conditions having similar symptomatology (DD and IBD) and HCs by analyzing dry plasma samples to identify a set of features that could be used for the univocal identification of CRC subjects differentiating them not only from healthy subjects but also from patients affected by different gastrointestinal disease presenting similar sintomatology. Raman spectra from dried plasma of CRC patients were also studied longitudinally at 6 days, 30 days, and 6 months after the surgery to assess the correlation between the spectral differences identified as characterizing the pathology and the effective presence of the disease, by monitoring the renormalization over time. Thanks to this work, we aim to develop a robust Raman method for identifying CRC from blood analysis. By focusing on selecting features that are effectively associated with the presence of CRC, we intend to avoid potential overfitting and the confounding effects of other gastrointestinal diseases.

## 2 RESULTS

### 2.1 Raman analysis of dried plasma of disease patients and HCs

Raman spectra from dry plasma was acquired from 120 subjects: 32 CRC patients, 37 IBD patients, 20 DD patients, and 31 HCs (Table 1).

**TABLE 1:** Clinical characteristics of the enrolled cohorts.

Sample	Number	Gender	Age, years, mean ± SD
HCs	31	16 M; 15F	68.22 ± 10.76
DD	20	14 M; 6F	60,55 ± 13.12
IBD	37	18 M; 19F	47.51 ± 10.76
CRC	32	17 M; 15F	69.72 ± 10.82

Clinical characteristics of the enrolled cohorts.

Abbreviations: HCs, healthy controls; DD, diverticular disease, IBD, inflammatory bowel disease such as Crohn's disease and ulcerative colitis; CRC, colorectal cancer.

**FIGURE 1:**
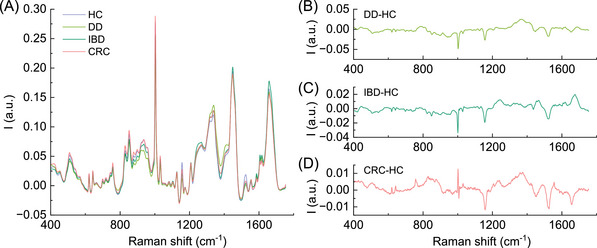
(A) Average normalized Raman spectra of all dry plasma samples from the different cohort of subjects included. HCs, light blue; DD, light green; IDB, dark green; CRC, light red. (B) differential spectra between DD and HCs; (C) differential spectra between IDB and HA; (D) differential spectra between DVD and HCs.

Clinical characteristics of the CRC patients are reported in Table S1. Figure 1A shows the overlay of the normalized average Raman spectra of the four different groups included in the study: CRC, HCs, DD, and IBD. To first highlight specific metabolic changes associated with the different conditions, the average spectrum of the dry plasma obtained from the HCs was subtracted from the average spectrum of each of the pathological classes. Figure 1B,D shows the difference spectra obtained by the subtraction.

Interestingly, the differential spectra of each disease seem to differ from each other, supporting the hypothesis that RS could be used to identify metabolic patterns specific of each condition. More in detail, a good degree of correlation was found between the differential spectra of IDB–HCs and DD–HCs (*R*_spearman_: 0.74). The differential spectrum of CRC–HCs is instead independent from both IDB–HCs (*R*_spearman_: 0.06) and DD–HCs (*R*_spearman_: 0.39).

Some of the peaks appear to be altered in all the groups. This is the case of carotenoids, whose main peaks at 1155 and 1524 cm^−1^ are clearly detected in the spectra, despite their relatively low plasmatic concentration, due to their conjugated, highly polarizable chemical structure.^33^

### 2.2 Analysis of Raman peaks referring to the different diseases

By comparing the spectral difference between the HCs and the pathological groups six main Raman bands were identified as divergent. In particular, the obtained results highlighted a more intense peak at 505 cm^−1^, corresponding to the presence of disulfide bridges in proteins,^34^ in CRC subjects. On the contrary, this peak was found less intense in DD and IBD subjects, confirming also a previous report on topic.^35^ A similar behavior (higher levels in CRC than in the other cohorts of included subjects) was found at the level of the peak related to aromatic amino acids such as tryptophan (760 cm^−1^) and the peak corresponding to the O‐P‐O of nucleic acids at 826 cm^−1^.^36^ Collectively, these variations identify differences distinguishing CRC from DD and IBD.

Inflammatory conditions were found to be characterized by statistically significant lower levels of the phenylalanine‐related peaks at 1003 cm^−1^,^36^, ^37^ which instead exhibited similar levels in CRC and HCs.

**FIGURE 2:**
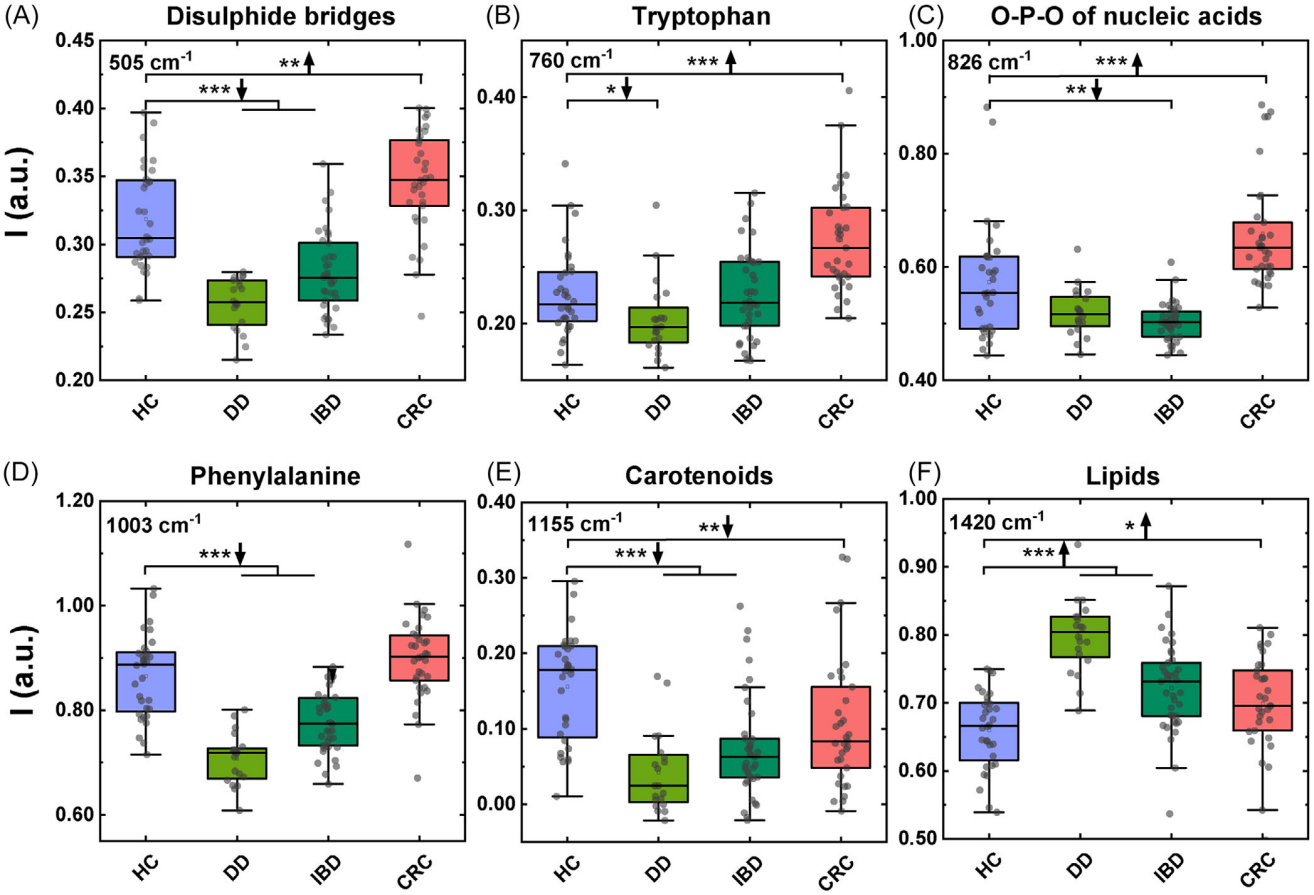
Box plot showing the different intensities of selected peaks between the different groups. Each data point represents an individual subject analysed. Each box represents the area between the 25th and 75th percentiles. Lines inside the boxes represent the median values. Upward arrows signify an increase, while downward arrows indicate a decrease in the intensities of selected peaks over the specified conditions DD, IBD and CRC compared with the HCs. Statistical significance: *p < 0.05; **p < 0.01; ***p < 0.0005.

Finally, the statistical analysis confirmed that some of the Raman peaks were altered in all the pathological conditions considered. In particular all subjects included in the CRC, DD, and IBD cohorts were characterized by a more intense peak related to plasma lipids at 1420 cm^−1^ and by lower peaks of carotenoids at 1155 and 1524 cm^−1^.^36^, ^38^ The box plots in Figure 2A–F show the statistical significance of the variations observed between the different groups for the peaks analyzed. The second peak of carotenoid at 1520 cm^−1^ was removed from the analysis as considered redundant with the one at 1155 cm^−1^ as they are both referring to the same class of metabolites they are in fact highly correlated with a Pearson's coefficient of 0.96 (Figure S1). The peak at 1155 cm^−1^ was thus chosen between the two because it is more narrow and because its shift and shape is less dependent from the contribution of the different carotenoids present in plasma.^33^, ^39^

Table 2 reports the statistical significance of the different peaks analyzed. Figure S2 report the mean value and the standard deviation observed for the six selected features on each of the subjects included in the study.

**TABLE 2:** Analysis of Raman peaks corresponding to the various diseases.

	Disulfide bridges in proteins (505 cm^−1^)	Tryptophan (760 cm^−1^)	O‐P‐O of nucleic acids (826 cm^−1^)	Phenylalanine (1003 cm^−1^)	Carotenoids (1155 cm^−1^)	Lipids (1420 cm^−1^)
HCs vs. DD	<0.0005	0.0103	n.s.	<0.0005	<0.0005	<0.0005
HCs vs. IBD	<0.0005	n.s.	0.0012	<0.0005	<0.0005	<0.0005
HCs vs. CRC	0.0044	<0.0005	<0.0005	n.s.	0.0062	0.0164
DD vs. IBD	0.0009	0.036	n.s.	<0.0005	n.s.	<0.0005
DD vs. CRC	<0.0005	<0.0005	<0.0005	<0.0005	0.003	<0.0005
CRC vs. IBD	<0.0005	<0.0005	<0.0005	<0.0005	n.s.	n.s.

Statistical analysis of the main Raman bands across different groups of patients. Shapiro–Wilk normality test was performed to check the normal distribution of data. *t*‐Test or Mann–Whitney test was performed based on results of the normality test.

Abbreviation: n.s., not significant.

### 2.3 Multivariate analysis of dried plasma Raman spectra from multiple diseases

**FIGURE 3:**
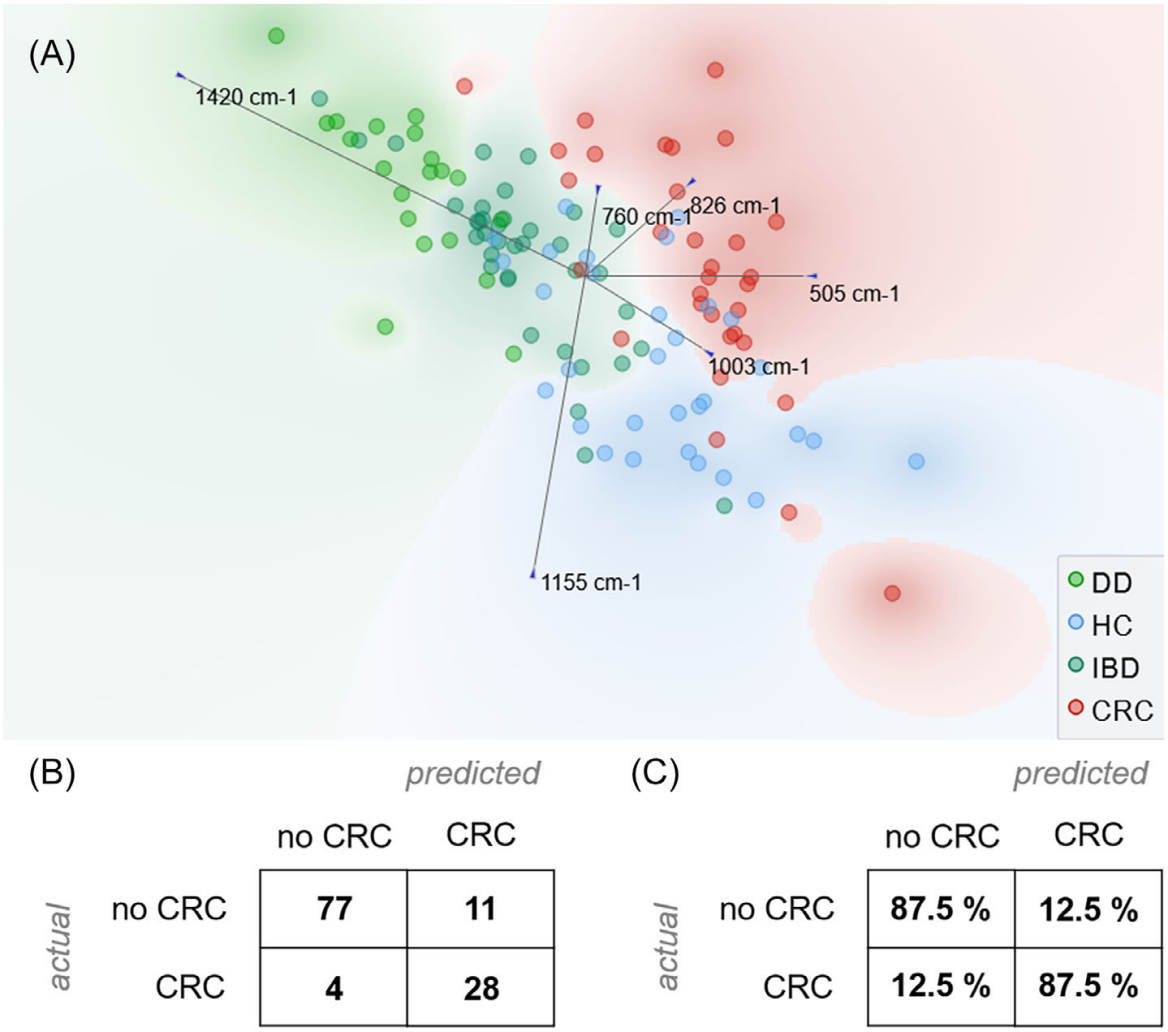
(A) Multivariate analysis of Raman spectra. Linear projection of the six characteristic features using the Freeviz algorithm. (B) The confusion matrix of the classification as number of patients. (C) The confusion matrix of the classification as proportion of actual patients.

A multivariate analysis of Raman spectra based on the identified features was performed to assess the potential of this approach in the identification of CRC patients versus other subjects (no CRC = HC + IBD + DD) (Figure 3). Figure 3A shows how a linear projection of the six identified features, based on a freeviz algorithm, can cluster the different diseases considered.^40^ To this purpose, the freeviz algorithm looks for the best linear projections that separate the data points according to their class, using a physical analogy of forces and springs. Freeviz highlights that the peaks related to tryptophan (760 cm^−1^) and carotenoids (1155 cm^−1^) primarily differentiate HCs from other groups. In contrast, disulfate bonds at 505 cm^−1^ are significant in distinguishing between IBD, DD, and CRC. The remaining selected peaks show variations across all considered groups.

A logistic regression model for the identification of CRC patients was then developed and validated using a leave‐one‐patient‐out approach. Logistic regression was chosen as a well‐established supervised machine learning algorithm widely used for binary classification tasks and that is commonly used for the analysis of biological Raman data.^41^, ^42^ The obtained results, after cross validation, show a good performance of RS with a classification accuracy of 87.5% and a F1 score of 0.879. Figure 3B,C reports the confusion matrix of the classification as number of patients and as proportion of actual patients. The same model was also applied to obtain a preliminary assessment of RS in the discrimination of the different clinical conditions as reported in Table S2.

It is notable to observe that among the four no‐CRC subjects that were misclassified, none of them had IBD or DD, supporting our claim that metabolic alterations induced by inflammation can be discriminated from those due to the presence of cancer.

### 2.4 Correlations with the clinical characteristics

The selected Raman features were investigated with clinical variables by Spearman correlation (Table 3).

**TABLE 3:** Spearman correlation analysis between the selected features and a clinical variable.

	Disulfide bridges in proteins (505 cm^−1^)	Tryptophan (760 cm^−1^)	O‐P‐O of nucleic acids (826 cm^−1^)	Phenylalanine (1003 cm^−1^)	Carotenoids (1155 cm^−1^)	Lipids (1420 cm^−1^)
BMI (ALL)	*ρ* = −0.04 *p* = 0.76	*ρ* = 0.21 *p* = 0.07	*ρ* = 0.09 *p* = 0.45	*ρ* = −0.01 *p* = 0.93	*ρ* = −0.17 *p* = 0.15	*ρ* = 0.23 *p* = 0.05
BMI (CRC)	*ρ* = −0.01 *p* = 0.96	*ρ* = 0.31 *p* = 0.09	*ρ* = 0.18 *p* = 0.33	*ρ* = −0.11 *p* = 0.54	*ρ* = −0.31 *p* = 0.09	*ρ* = 0.34 *p* = 0.06
CEA (CRC)	*ρ* = −0.13 *p* = 0.48	*ρ* = 0.32 *p* = 0.08	*ρ* = −0.10 *p* = 0.61	*ρ* = −0.23 *p* = 0.22	***ρ* = −0.52** ***p* = 0.003**	*ρ* = 0.23 *p* = 0.22

Results of the Spearman correlation analysis. Bold: statistically significant Spearman correlation

To exclude a possible association of increased intensity in lipid spectra with obese or overweight patients, the body mass index (BMI) was considered for the correlation analysis. 1420 cm^−1^ peak and BMI status showed a positive trend of correlation but the test did not reach the statistical significance, suggesting that the modulation of lipid peak was independent from subject lipid variability. Given that the cohort of patients with IBD and DD was younger than those with HCs and CRC (see Table 1), we employed a generalized linear model^43^ to analyze the effect of age on the differences between groups. This model allowed us to determine whether the mean peaks among groups remained distinct when accounting for both disease and age simultaneously (Table S3). The results confirmed that the statistically significant differences persisted even after considering the effect of age, consistent with the differences observed without accounting for age.

On the subgroup of patients affected by CRC (*n* = 32), the relationship between the CEA levels before the surgical treatment and the six selected Raman peaks was also studied. In this case, the data obtained showed an inverted correlation only with the 1155 cm^−1^ peak related to the carotenoids.

### 2.5 Postoperative monitoring of the CRC by RS

**FIGURE 4:**
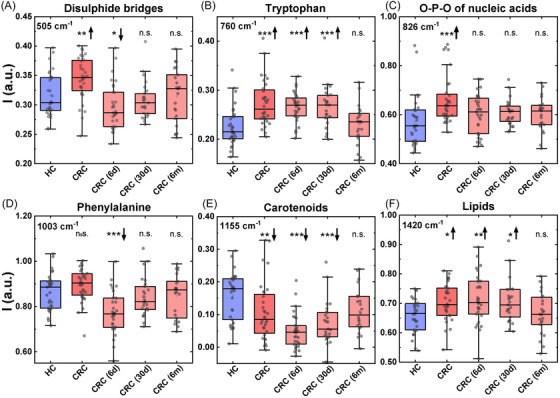
Box plots showing the different Raman intensity of selected peaks in CRC patients versus HCs over the time. Data are shown as box and whisker plots. CRC, preoperative patients; 6 days (6d), 30 days (30d), and 6 months (6 m) CRC patients after the surgery treatment. Each box represents the area between the 25th and 75th percentiles. Lines inside the boxes represent the median values. Upward arrows signify an increase, while downward arrows indicate a decrease in the intensities of selected peaks over HCs. Statistical significance: *p < 0.05; **p < 0.01; ***p < 0.0005.

The Raman biochemical profile of the six peaks in CRC patients was then evaluated longitudinally 6 days, 30 days, and 6 months after surgery (Figure 4A–F). These spectra were compared with those obtained from the control group of HCs, to observe if the Raman fingerprint of CRC patients normalized during the follow‐up. At day 6, we observed different pattern of modulation in the RS profile compared with patients with CRC. While the RS shifts observed at day 6 can potentially be ascribed to the postsurgical period, the modulation occurred after 6 months suggest a normalization toward healthy condition following tumor removal. In particular, the significant upregulation of the peak intensity associated with disulfide bridge (Figure 4A), O‐P‐O of nucleic acid (Figure 4C) and lipids (Figure 4F) observed in CRC was initially reduced after 30 days from surgery and then further decreased 6 months after surgery, displaying Raman intensities comparable to HCs. In contrast, the peak related to carotenoids (Figure 4E), which was significantly decreased in the presence of CRC, increased starting from 30 days after surgery up to 6 months after tumor removal, reaching levels that were not statistically different from the healthy condition. The results obtained at 6 months were also used as test set for the classification algorithm previously reported resulting in a low classification accuracy of 47.8% and a F1 score of 0.647. This result further proves the slow renormalization of the Raman profile of the patients after surgery and imply that some of them still results altered and support for the further Raman monitoring of CRC at even longer times.

## 3 DISCUSSION

A specific CRC biomarker both for diagnosis and disease monitoring is still an unmet clinical need. RS represents a noninvasive application uncovering circulating molecular profiling and could help in the differential diagnosis among colon diseases. To test our hypothesis, we focused on acquiring high‐quality Raman spectra from dried plasma samples of patients with different gastric conditions. While it is possible to acquire Raman spectra from liquid plasma,^44^ the use of dried plasma spots allowed us to obtain spectra with a better signal‐to‐noise ratio. This improvement in signal quality enabled a more accurate assessment of differences among multiple classes of biomolecules. Specifically, we utilized a 785 nm laser for our Raman measurements to avoid resonance effects on specific classes of biomolecules, such as carotenoids. This choice allows us to capture an overview of the concentrations of multiple classes of biomolecules, including proteins, nucleic acids, and lipids, in a single measurement.

To the best of our knowledge, this work compared for the first time Raman peaks of dried plasma samples from a broad spectrum of different bowel diseases with a common low‐grade chronic inflammation. Interestingly, we found specific biomolecular spectral pattern associated with CRC. Those spontaneous RS shifts could reflect alterations in metabolic demand and identify plasma metabolites unique to CRC onset, thereby providing evidence that a single blood test might accurately intercept CRC patients requiring colonoscopy.

Specifically, CRC patients showed enrichment of Raman intensities at 505, 760, and 826 cm^−1^ originated from disulfide bridges in proteins, presence of the aromatic amino acid tryptophan and O‐P‐O nucleic acids, respectively. This profile may reflect the increasing rate of protein synthesis and DNA double‐strand breakage occurring during CRC epithelial proliferation.^45^, ^46^ and mirror the increase of metabolites known to counteract antitumor immunosurveillance and tumor progression.^47^ Further, after 6 months follow‐up CRC patients showed a normalization of the six peaks, similar to HCs. This suggests that the presence or absence of the tumor could be detected by specific RS fingerprint, indicating that this approach could be a novel tool for CRC monitoring.

One limitation of this study is the relatively low number of subjects involved, making it a preliminary investigation. The sample size was insufficient to definitively validate the diagnostic performance of RS in discriminating CRC patients. However, the promising results support the need for further analysis. Another limitation was the exclusion of patients with colorectal adenomas. Indeed, the present study was aimed at finding a Raman signature predictive of CRC to be used in a cohort of patients with gastrointestinal symptoms, rather than a wide screening tool for asymptomatic patients. Since CRC, DD, and IBD often share similar symptoms, including bleeding, abdominal pain, occlusion, and so on, Raman spectra from these cohorts of patients were included. Conversely, colorectal adenomas are often incidentally found in screening colonoscopies performed after positive fecal occult blood test, and very rarely adenomas are symptomatic. Anyway, the inclusion of colorectal adenomas could have refined the Raman signature providing significant data. At last, it must be noticed that although RS shows great promises in the diagnostic field, there is still no standardized and universally acceptable protocol for the analysis of biofluids. For now, this problem still limits the use or RS at a research level and remain to be addressed before a clinical implementation of this promising method.^48^

## 4 CONCLUSIONS

In summary, RS on dried plasma samples emerges as a viable, cost‐effective technique that requires no specific sample processing, thereby holding significant translational potential. Our current results indicate that RS stands out as a potentially effective tool to identify CRC patients using a liquid biopsy approach. This would avoid the need for unnecessary colonoscopies, mitigating associated adverse effects and related costs for nationals healthcare systems. Furthermore, our data, demonstrating the renormalization of the identified specific RS features after surgery, underscore the potential for RS to serve as a reliable method for monitoring CRC during patients’ follow‐up, unlocking the utility of RS in enhancing disease surveillance and management.

## 5 MATERIALS AND METHODS

### 5.1 Patient selection

Patients aged >18 years old and with a proven diagnosis of DD (*n* = 20) and IBD (*n* = 37) were referred to the Unit of Inflammatory Bowel Disease of L. Sacco Hospital, Milan and involved in the study under the protocol n. 24916/2019 approved by the Ethical Committee of Milano Area 1. Recruited DD patients included subjects with absence of acute symptoms, any Hinchey stage, documented by colonoscopy, and/or CT abdomen with contrast medium. IBD patients included subjects with Crohn's disease and ulcerative colitis, in outpatient monitoring for the pathology or with surgical indication, with no other concurrent gastrointestinal and/or autoimmune diseases. All the patients were in pharmacological washout at the time of enrollment. A control group of 31 HCs, free from any type of symptomatology and/or clinical‐diagnostic finding of a gastro‐intestinal, rheumatological, and/or oncological pathology, was also enrolled within the same protocol. A prospective cohort of 32 consecutive patients with CRC and treated at the Colorectal Surgery Unit of Fondazione IRCCS, Istituto Nazionale dei Tumori, Milan between 2019 and 2021 for up front surgery were accrued in this study under the protocol n. INT127/19. In the period between March 2020 to January 2021, no patient was accrued due to COVID lockdowns and related interruptions in research activities. Recruited CRC patients included subjects aged >18 years old, with T2–T4, any N stage and microsatellite stability status. All CRC cases were defined by the endoscopic diagnosis of a colorectal lesion with histologically proven adenocarcinoma on biopsies, and with T2–T4, any N stage observed at computed tomography scans.

Patients treated with immunosuppressive medication or neoadjuvant chemotherapy within the last 6 months prior surgery were excluded from the study. The study protocols were approved by the Ethical Committee and conducted in compliance with the Declaration of Helsinki. All the patients and healthy subjects included in this study provided written and informed consent. The main clinical characteristics of the study cohorts are summarized in Table S1.

### 5.2 Blood collection

Peripheral venous blood was collected from each subject in K2EDTA‐coated vacutainer tubes. Plasma samples were obtained by whole blood centrifugation (1300×*g* × 10 min) at room temperature, repeated two times. Plasma was aliquoted and stored in sterile cryovials at −80°C until analysis.^35^

### 5.3 Raman spectra acquisition and analysis 

Raman spectra were acquired using an InVia Reflex confocal Raman microscope (Renishaw, Wootton‐under‐Edge, UK) equipped with a laser light source operating at 785 nm. The Raman spectrometer was calibrated before any acquisition session using the 520.7 cm^−1^ band of a silicon wafer.

In a typical experiment, a 4‐µL drop of plasma was deposited on the surface of CaF_2_ discs (Crystran, Poole, UK) and dried for 20 min at room temperature under laminar flow. The Raman spectra acquisition was performed using a 785‐nm laser light source with 100% power (around 90 mW at source), a 1200 L/mm grating, and a 100× objective. The illuminate area in each measure is about 2 µm in diameter. Spectra were acquired in the region between 400 and 1800 cm^−1^ as the sum of seven acquisitions of 10 s each. Spectrum resolution is about 1.1 cm^−1^. For each sample, three different spectra were collected from different positions of the drop. Laser focus on the sample was monitored before each acquisition. The spots were collected on the outer region of the dried drop that allows to minimize the difference due to the inhomogeneous distribution of the biomolecules during the drying process.^49^

The software WIRE 5 (Renishaw, UK) was used for the spectral acquisition and to remove cosmic rays. Background signal was removed using an Asymmetric Least Square Smoothing Baseline with a 2th order polynomial fit. Each Raman spectra was normalized dividing the intensity measured at each Raman shift for the calculated area of the spectral region between 1434 and 1440 cm^−1^. The average of the three spectra acquired on the different position of the drop was regarded as the representative spectrum of each patient. Data analysis was performed using OriginPro 2019 (OriginLab, Northampton, MA, USA). The peaks present in the Raman spectra refer to different functional groups present that can be ascribed at specific classes of biomolecules.^30^, ^35^, ^36^, ^37^, ^50^ Variations in the intensity of the peak intensity were used to infer the relative variation in the amount of each biomolecule in the plasma. Table S4 reports the spectral windows used to identified the selected features. Table S5 reports a complete attribution of the main Raman peaks present in the spectra of dried plasma, based on the relevant scientific literature on the topic.^30^, ^34^, ^36^, ^37^, ^38^, ^51^, ^52^, ^53^, ^54^, ^55^, ^56^, ^57^, ^58^, ^59^, ^60^

### 5.4 Statistical data analysis

For the statistical analysis of data, the Shapiro–Wilk test was first applied to each group to verify the normal distribution of data; data were considered as normal only if the test accepted the null hypothesis of normal distribution. Subsequently, parametric (*t*‐test) or nonparametric (Mann–Whitney test, Wilcoxon test) tests were applied as appropriate to compare mean values between the different groups. To determine if age affects differences in Raman peaks, we computed a generalized linear model for each peak, incorporating age as a variable across different patient groups.

To reduce the influence of noise on the data analysis, the peak intensity was calculated as the average value of a band around the specific peak.

Statistical significance was set at *p* value < 0.05. Multivariate data analysis of the spectra was performed using Quasar,^61^ an open‐source software for bio‐spectroscopy. The freeviz algorithm with random initialization and gravity 0.1 was used to identify the contribution of the different features to the discrimination. The logistic regression widget was used for patient classification without any data regularization and results were cross validated by leave one patient out approach.

## AUTHOR CONTRIBUTIONS

*Study concept and design*: Fabio Corsi, Carlo Morasso, Luca Sorrentino, Licia Rivoltini, and Elena Daveri. *Acquisition of Raman spectra*: Carlo Morasso and Arianna Bonizzi. *Statistical analysis*: Carlo Morasso, Sara Albasini, and Arianna Bonizzi. *Sample collection and preparation*: Elena Daveri and Sara Albasini. *Analysis and interpretation of data*: Carlo Morasso, Sara Albasini, Elena Daveri, Marta Truffi, and Serena Mazzucchelli. *Selection and recruitment of subjects included in the study*: Luca Sorrentino, Francesco Colombo, Marta Truffi, Licia Rivoltini, and Piergiorgio Danelli. *Study supervision*: Fabio Corsi. All authors have read and approved the final manuscript.

## CONFLICT OF INTEREST STATEMENT

The authors disclose no conflict of interest.

## ETHICS STATEMENT

This study was approved by the Ethical Committee of Milano Area 1 (Approved No: 24916/2019) and Fondazione IRCCS, Istituto Nazionale dei Tumori, Milan (Approved No: INT127/19). All the patients and healthy subjects included in this study provided written and informed consent.

## Supporting information



Supporting Information

## Data Availability

The datasets used and analyzed during the current study are available from the corresponding author on reasonable request.
