# Supplementary material for: Raman spectroscopy on dried blood plasma allows diagnosis and monitoring of colorectal cancer
Source: MedComm (2020). 2024 Oct 31;5(11):e774. doi: 10.1002/mco2.774 (PMC11527808; doi:10.1002/mco2.774)
Supplement: Supplementary file 1 — Supporting Information [file MCO2-5-e774-s001.docx]

Supplementary Information

**Raman Spectroscopy on dried plasma allows diagnosis and monitoring of Colorectal Cancer**

**Authors**

Carlo Morasso^1^; Elena Daveri^2^; Arianna Bonizzi^1^; Marta Truffi^1^; Francesco Colombo^3^; Piergiorgio Danelli^3,5^; Sara Albasini^4^; Licia Rivoltini^2^; Serena Mazzucchelli^5^; Luca Sorrentino^6^; Fabio Corsi^4,5^

1. Laboratory of Nanomedicine, Istituti Clinici Scientifici Maugeri IRCCS, Pavia, Italy
2. Translational Immunology Unit, Fondazione IRCCS Istituto Nazionale dei Tumori di Milano, Milan, Italy
3. Division of General Surgery, "Luigi Sacco" University Hospital, ASST Fatebenefratelli-Sacco, Milan, Italy
4. Breast Unit, Istituti Clinici Scientifici Maugeri IRCCS, Pavia, Italy
5. Department of Biomedical and Clinical Sciences, University of Milan, Milan, Italy
6. Colorectal surgery unit, Fondazione IRCCS Istituto Nazionale dei Tumori di Milano, Milan, Italy

^*^ Correspondence: Fabio Corsi, Breast Unit, Istituti Clinici Scientifici Maugeri IRCCS, Pavia, Italy; Department of Biomedical and Clinical Sciences, University of Milan, Milan, Italy. Email: fabio.corsi@icsmaugeri.it

**Table S1:** Characteristics of patients with Colorectal Cancer

| **Clinical and pathological variables** | **Value** |
| --- | --- |
|  |  |
| **CEA (ng/mL)** | 8.2 (18.1) |
| **CA19.9 (U/mL)** | 14.6 (17.1) |
|  |  |
| **Cancer localization** |  |
| Ascending colon | 9 (28.1%) |
| Transverse colon | 3 (9.4%) |
| Descending/sigmoid colon | 10 (31.2%) |
| Upper rectum | 10 (31.2%) |
|  |  |
| **Symptomatic bowel occlusion** |  |
| Yes | 3 (9.4%) |
| No | 29 (90.6%) |
|  |  |
| **pT stage** |  |
| pT2 | 9 (28.1%) |
| pT3 | 19 (59.4%) |
| pT4 | 4 (12.5%) |
|  |  |
| **pN stage** |  |
| pN0 | 23 (71.9%) |
| pN1 | 7 (21.9%) |
| pN2 | 2 (6.2%) |
|  |  |
| **Distant metastases** |  |
| Yes | 0 (0.0%) |
| No | 32 (100.0%) |
|  |  |
| **Grading** |  |
| G1-G2 | 25 (78.1%) |
| G3 | 7 (21.9%) |
|  |  |
| **Microsatellite status** |  |
| MSS | 29 (90.6%) |
| MSI-H | 3 (9.4%) |


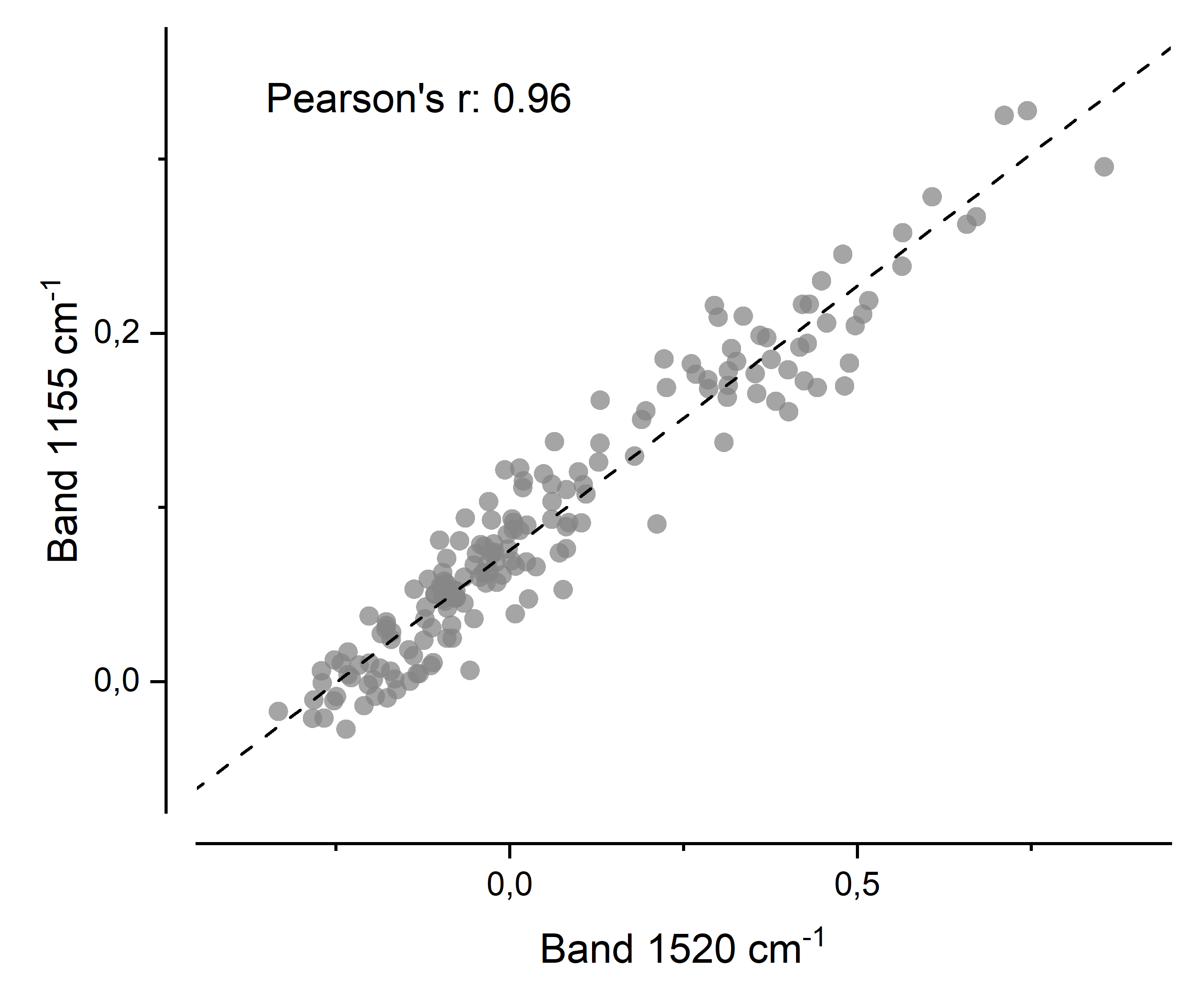


**Figure S1:** Correlation between the two bands relative to carotenoids at 1155 and 1520 cm-1.

| 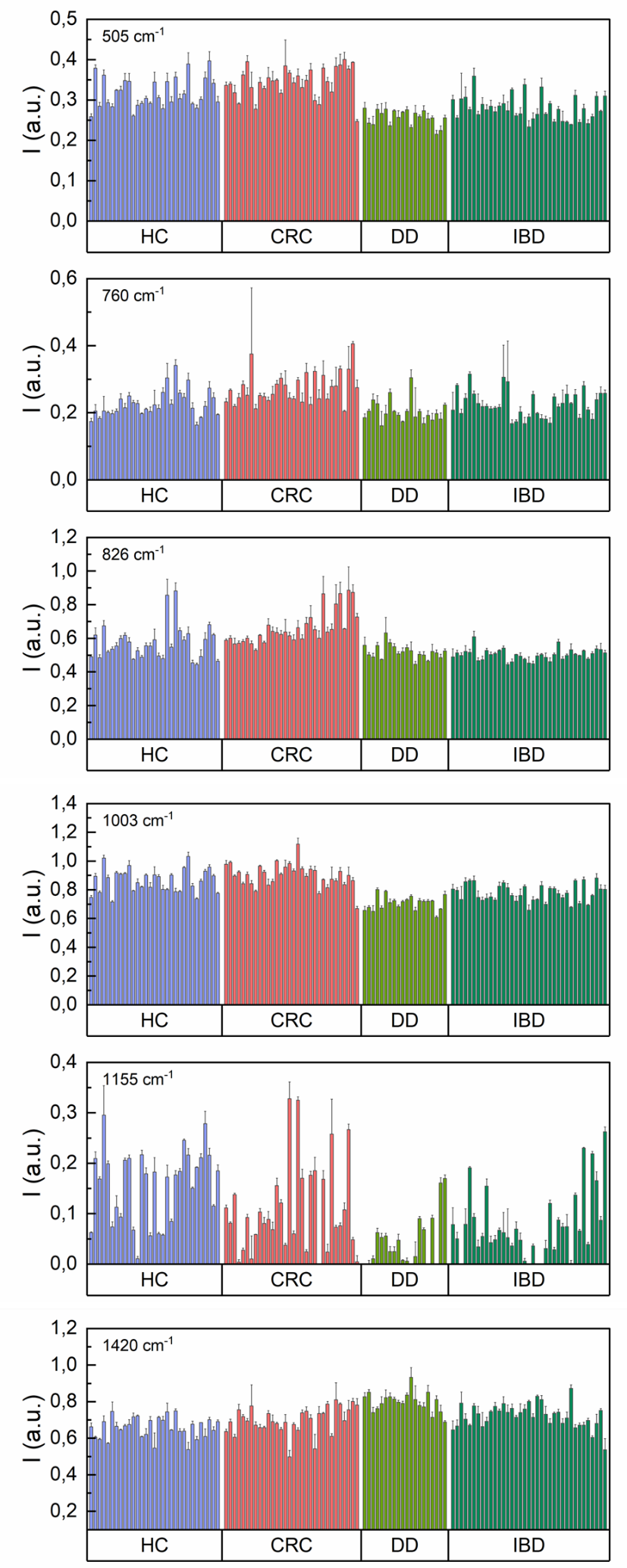 |
| --- |

**Figure S2:** Mean value and standard deviation of the six considered features for each of the subjects included in the study

**Table** **S2**: results of the logistic regression classification of the different classes of subjects included

| **Class A** | **Class B** | **n Class A** | **n Class B** | **Classification Accuracy** | **F1 score** |
| --- | --- | --- | --- | --- | --- |
| **CRC** | **HC** | 32 | 31 | 0.746 | 0.750 |
| **IBD** | **HC** | 37 | 31 | 0.809 | 0.831 |
| **DD** | **HC** | 20 | 31 | 0.922 | 0.900 |
| **CRC** | **IBD** | 32 | 37 | 0.928 | 0.921 |
| **DD** | **IBD** | 20 | 37 | 0.807 | 0.718 |
| **CRC** | **DD** | 32 | 20 | 0.962 | 0.968 |

**Table S3:** generalized linear model reporting the effect of age on the difference of the features among classes

|  | **505 cm^-1^** | | | **760 cm^-1^** | | | **826 cm^-1^** | | |
| --- | --- | --- | --- | --- | --- | --- | --- | --- | --- |
|  | **β** | **95%CI** | **p** | **β** | **95%CI** | **p** | **β** | **95%CI** | **p** |
| **Age** | 0.0001 | -0.0005;0.0006 | 0.87 | 0.0003 | -0.0003;0.001 | 0.29 | 0.0003 | -0.0009;0.001 | 0.60 |
| **DD** | -0.09 | -0.11;-0.07 | <0.0001 | -0.07 | -0.09;-0.04 | <0.0001 | -0.14 | -0.18;-0.09 | <0.0001 |
| **HC** | -0.03 | -0.04;-0.01 | 0.001 | -0.05 | -0.07;-0.03 | <0.0001 | -0.09 | -0.12;-0.05 | <0.0001 |
| **IBD** | -0.06 | -0.08;-0.04 | <0.0001 | -0.04 | -0.06;-0.02 | 0.0009 | -0.15 | -0.19;-0.11 | <0.0001 |
| **CRC** | Ref. |  |  | Ref. |  |  | Ref. |  |  |
|  | **1003 cm^-1^** | | | **1155 cm^-1^** | | | **1420 cm^-1^** | | |
|  | **β** | **95%CI** | **p** | **β** | **95%CI** | **p** | **β** | **95%CI** | **p** |
| **Age** | 0.0005 | -0.0006;0.002 | 0.38 | 0.0003 | -0.0009;0.001 | 0.67 | 0.0004 | -0.0006;0.001 | 0.38 |
| **DD** | -0.19 | -0.22;-0.15 | <0.0001 | -0.07 | -0.10;-0.02 | 0.003 | 0.10 | 0.07;0.14 | <0.0001 |
| **HC** | -0.03 | -0.07;-0.0005 | 0.05 | 0.05 | 0.01;0.08 | 0.009 | -0.04 | -0.07;-0.01 | 0.01 |
| **IBD** | -0.11 | -0.15;-0.07 | <0.0001 | -0.03 | -0.07;0.01 | 0.21 | 0.03 | -0.004;0.07 | 0.08 |
| **CRC** | Ref. |  |  | Ref. |  |  | Ref. |  |  |

**Table S4.** Spectral measurement ranges for peaks analyzed

| **Peak Position (cm-1)** | **Range (cm-1)** | **Assignment** |
| --- | --- | --- |
| 505 cm-1 | 505-512 cm-1 | Disulphide bridges |
| 760 cm-1 | 754-764 cm-1 | Tryptophan |
| 826 cm-1 | 819-832 cm-1 | Tyrosine/protein |
| 1003 cm-1 | 1000-1005 cm-1 | Phenylalanine |
| 1155 cm-1 | 1153-1159 cm-1 | Carotenoids |
| 1420 cm-1 | 1417- 1429 cm-1 | Lipids |

**Table S5.** Main Raman peaks present in blood plasma

| **Peak Position (cm-1)** | **Assignment** | **Ref.** |
| --- | --- | --- |
|  |  |  |
| 505 | Disulphide bridges in proteins | Nakamura et al,, 1997 |
| 570 | Tryptophan/cytosine, guanine | Stone et al,, 2004 |
| 627 | Carbohydrates | De Gelder et al,, 2007 |
| 644 | C-C twisting mode of Tyrosine | Stone et al,, 2004 |
| 700 | Cholesterol | Bonizzi et al,, 2023 |
| 718 | Choline group | Krafft et al., 2005 |
| 760 | Tryptophan | Shetty et al., 2006; Talari et al., 2015 |
| 828 | O-P-O stretching RNA | Stone et al,, 2004 |
| 840 | Carbohydrates | Stone et al,, 2004 |
| 848 | Ring breathing of tyrosine and C-C stretch of proline ring | Stone et al., 2004 |
| 878 | Tryptophan | Talari et al., 2015 |
| 901 | Monosaccharides (b-glucose), (C-O-C) skeletal | Shetty et al., 2006 |
| 960 | *Trans* C=C | Bonizzi et al., 2023 |
| 1003 | Phenylalanine | Poon et al., 2012 |
| 1032 | C–H in-plane bending mode of phenylalanine | Poon et al., 2012 |
| 1082 | C–N stretching mode | Poon et al., 2012 |
| 1100 | C-C vibration mode of the gauche-bonded chain | Huang et al.,2005 |
| 1125 | C–C stretching mode of lipids/protein C–N stretch | Stone et al., 2004 |
| 1157 | Carotenoids | Casella,et al., 2011 |
| 1173 | Cytosine, guanine | Ruiz-Chica et al., 2004 |
| 1208 | Tryptophan and phenylalanine n(C–C_6_H_5_) mode | Poon et al., 2012 |
| 1270 | Amide III; C=C lipids groups in unsaturated fatty acids | Mahadevan-Jansen, et al., 1996; Krafft et al., 2005 |
| 1300 | Triglycerides | Bonizzi et al., 2023 |
| 1316 | Lipids | Stone et al., 2004 |
| 1330 | Tryptophan | Rygula et al., 2017 |
| 1339 | DNA ; Carb | Vanna et al., 2024 |
| 1399 | C=O symmetric stretch | Faoláin et al., 2005 |
| 1420 | CH_2_ scissoring vibration (lipid band); CH_2_ bending mode of proteins and lipids | Talari et al., 2015 |
| 1448 | Protein/lipids CH2 bending | Faoláin et al., 2005 |
| 1524 | Carotenoids | Casella,et al., 2011 |
| 1552 | Tryptophan | Poon et al., 2012 |
| 1604 | Ring C-C Phenylalanine | Stone et a, 2004 |
| 1616 | Tryptophan and Tyrosine v(C=C) | Poon et al., 2012 |
| 1656 | *Cis* C=C | Bonizzi et al., 2023 |
| 1675 | Amide I | Faoláin et al., 2005 |
| 1742 | Lipids | Talari et al., 2015 |

**References used for the peak assignment:**

1. Nakamura, K. *et al.* Conformational changes in seventeen cystine disulfide bridges of bovine serum albumin proved by Raman spectroscopy. *FEBS Lett.* **417**, 375–378 (1997).
2. Stone, N., Kendall, C., Smith, J., Crow, P. & Barr, H. Raman spectroscopy for identification of epithelial cancers. *Faraday Discuss.* **126**, 141 (2004).
3. De Gelder, J., De Gussem, K., Vandenabeele, P. & Moens, L. Reference database of Raman spectra of biological molecules. *J. Raman Spectrosc.* **38**, 1133–1147 (2007).
4. Bonizzi, A. *et al.* Determination of the quality of lipoproteins by Raman spectroscopy in obese and healthy subjects. *The Analyst* **148**, 2012–2020 (2023).
5. Krafft, C., Neudert, L., Simat, T. & Salzer, R. Near infrared Raman spectra of human brain lipids. *Spectrochim. Acta. A. Mol. Biomol. Spectrosc.* **61**, 1529–1535 (2005).
6. Shetty, G., Kendall, C., Shepherd, N., Stone, N. & Barr, H. Raman spectroscopy: elucidation of biochemical changes in carcinogenesis of oesophagus. *Br. J. Cancer* **94**, 1460–1464 (2006).
7. Talari, A. C. S., Movasaghi, Z., Rehman, S. & Rehman, I. U. Raman Spectroscopy of Biological Tissues. *Appl. Spectrosc. Rev.* **50**, 46–111 (2015).
8. Poon, K. W. C. *et al.* Quantitative reagent-free detection of fibrinogen levels in human blood plasma using Raman spectroscopy. *The Analyst* **137**, 1807 (2012).
9. Huang, Z., Lui, H., McLean, D. I., Korbelik, M. & Zeng, H. Raman Spectroscopy in Combination with Background Near‐infrared Autofluorescence Enhances the *In Vivo* Assessment of Malignant Tissues. *Photochem. Photobiol.* **81**, 1219–1226 (2005).
10. Casella, M. *et al.* Raman and SERS recognition of β-carotene and haemoglobin fingerprints in human whole blood. *Spectrochim. Acta. A. Mol. Biomol. Spectrosc.* **79**, 915–919 (2011).
11. Ruiz‐Chica, A. J., Medina, M. A., Sánchez‐Jiménez, F. & Ramírez, F. J. Characterization by Raman spectroscopy of conformational changes on guanine–cytosine and adenine–thymine oligonucleotides induced by aminooxy analogues of spermidine. *J. Raman Spectrosc.* **35**, 93–100 (2004).
12. Mahadevan-Jansen, A. Raman spectroscopy for the detection of cancers and precancers. *J. Biomed. Opt.* **1**, 31 (1996).
13. Rygula, A. et al. Raman spectroscopy of proteins: a review. *J. Raman Spectrosc*. **44**, 1061–1076 (2013).
14. Vanna, R. *et al.* High-Resolution Raman Imaging of >300 Patient-Derived Cells from Nine Different Leukemia Subtypes: A Global Clustering Approach. *Analytical Chemistry* **96**, 9468–9477 (2024).
15. Ó Faoláin, E. *et al.* A study examining the effects of tissue processing on human tissue sections using vibrational spectroscopy. *Vib. Spectrosc.* **38**, 121–127 (2005).
